# Optical Dispersions of Bloch Surface Waves and Surface Plasmon Polaritons: Towards Advanced Biosensors

**DOI:** 10.3390/ma12193147

**Published:** 2019-09-26

**Authors:** Zigmas Balevicius, Algirdas Baskys

**Affiliations:** 1 Faculty of Electronics, Vilnius Gediminas Technical University, Naugarduko st. 41, 03227 Vilnius, Lithuania; algirdas.baskys@ftmc.lt; 2 State Research Institute Center for Physical Sciences and Technology, Saulėtekio av. 3, LT-10257 Vilnius, Lithuania

**Keywords:** bloch surface waves, surface plasmon polariton, optical biosensors, photonic crystals, silicon oxide, titanium oxide

## Abstract

The total internal reflection ellipsometry (TIRE) method was used for the excitation and study of the sensitivity features of surface plasmon polariton (SPP) and Bloch surface waves (BSWs) resonances. For the BSWs generation distributed Bragg gratings were formed on the tops of the substrates (BK7 glass substrate), which had six bilayers of ~120 nm SiO_2_ and ~40 nm TiO_2_ and 40 nm of TiO_2_ on the top. The SPP sample consisted of the BK7 glass prism and a gold layer (45 nm). Numerical calculations of the optical dispersions and the experimental TIRE data have shown that SPP resonance overtake the BSWs in wavelength scanning by a factor of about 17. However, for the ellipsometric parameters *Ψ* and *Δ* in the vicinity of excitations, the BSW sensitivity is comparable with SPP. The obtained resolutions were $\Delta_{SPP}=7.14\times 10^{-6}RIU$, $\Psi_{SPP}=1.7\times 10^{-5}RIU$ for the SPP and $\Delta_{BSW}=8.7\times 10^{-6}RIU$, $\Psi_{BSW}=2.7\times 10^{-5}RIU$ for the BSW. The capabilities of both surface excitations are discussed from the sensitivity point of view in the design of these advanced biosensors.

## 1. Introduction

Bloch surface waves (BSWs) are the type of surface electromagnetic waves, which can be excited on the surfaces of photonic crystals (PC) [1]. A lot of attention has been paid to the application of such BSWs for optical sensing and integrated photonic devices [2]. The BSWs exist at the interface between the periodic dielectric structure with a distributed Bragg grating and the surrounding medium. Due to their optical dispersion features, the excitation of BSW can be tuned along a wide spectral range by changing the PC materials and the period of the bilayers [3]. It has been shown that BSW are suitable for fluorescence sensing applications. Being dielectrics, their surfaces do not quench the signal intensity as happens in the case of metals [4]. The biocompatibility of dielectric surfaces with biomolecules, immune-sensing and other life science applications make the BSW an attractive tool for optical surface sensing, which can replace the very widely spread use of surface plasmon resonance sensors [5]. Surface plasmon polariton (SPP) optical sensors are still the most popular due to the high sensitivity which can be obtained because of the dielectric permittivity dispersion features of metals and the enhancement of the electric field at the metal/dielectric interfaces [6,7]. In SPP-type optical sensors, a glass prism is commonly used as a coupler to achieve conditions of total internal reflection (TIR) and to excite the propagated SPP waves, which are transverse magnetic (TM) p-polarized. The BSW, however, can be generated in both TM (p-) and TE (s-) polarizations, which, in some cases, allow the monitoring of the events at the surface in both polarizations.

The total internal reflection (TIR) phenomenon is one of the more common optical configurations used in biosensor design [6]. This makes possible the monitoring of biomolecule immobilization processes on their surfaces and their interaction with other proteins in opaque media. Moreover, the exploitation of such evanescent surface waves allows one to avoid the propagation of light directly through the liquids, which induces additional signal noises and decreases sensitivity [7]. TIR setups are also used in surface plasmon resonance (SPR) [8] and BSW based biosensors [3]. The SPP and the BSW can be generated by using a glass prism with a gold layer (SPP) or with a PC structure (BSW) attached to its base. Both resonances can be excited at an appropriate angle of incidence (AOI) to the prism, the SPP due to the matching of the SPP in-plane wave vector [5] and the BSW due to the in-plane wave vector in the photonic stop band of the PC [3]. However, a detailed analysis of TM and TE polarizations can be obtained by employing the spectroscopic ellipsometry technique in its total internal reflection geometry (TIRE) [9]. The TIRE method combines the full analysis of the polarization properties in ellipsometry and increases the sensitivity of the analyzed surfaces by generating SPPs on the gold or BSWs on the surface of the PC. Moreover, the possibility of monitoring these processes in real time, non-destructively and conducting the analysis of their kinetic curves is very important in real bio-sensing applications [10], as well as for optical gas-sensing [11,12].

The dielectric (PC)/dielectric interface has some advantages for biosensing applications when compared with the metal/dielectric interface. Its biocompatibility and the low toxicity of the dielectrics enable one to ensure suitable conformational states of the proteins on the sensing surface, which is very important in cancer biomarkers [13], immune-sensing [14] and other biomedical applications [15]. Thus, the possibilities of using dielectric interfaces instead of metal ones is very attractive.

During the last decade, much attention has been given to the analysis of the comparative sensitivity of such BSW and SPR optical sensors [3,16]. A number of publications have been dedicated to applications of BSWs for bio- and gas sensing due to their better electric field enhancement and the lower losses that are obtained than when using the SPP [17,18]. Total internal reflection configurations with prism couplers have been widely used for photonic crystal applications in optical sensing [19,20]. In some works, single wavelength ellipsometry in internal reflection setups were applied for the employment of the phase properties of BSWs in sensing applications [21,22]. The increased sensitivity of the BSW sensing properties with phase registration are shown [23]. It should be noted, that better phase sensitivity over amplitude were also shown earlier for SPP waves, employing the ellipsometry method [9,24]. However, the comparative analysis of the dispersion relations of BSW and SPP methods have not been done before from the sensitivity point of view. In this sense, TIRE in its spectroscopic mode is a very suitable method.

The aim of these studies has been to investigate the sensitivity properties of surface plasmon polaritons and Bloch surface waves by utilizing TIRE configurations of spectroscopic ellipsometry, thus revealing the behaviors of both surface excitations, which were determined from the dispersion relation features at the interfaces. The capabilities of both surface excitations are discussed from the sensitivity point of view in the design of these advanced biosensors.

## 2. Materials and Methods

A sample with photonic crystal structure was produced by using an ion beam sputtering (IBS) plant. Before the deposition process, the vacuum chamber was held at 50 °C heat for 1 h. To remove the impurity layer, the ion source was used for pre-sputtering of the target before deposition. During the process, oxygen gas was supplied toward the substrate to ensure complete oxidation of the growing coating. A radio-frequency grid-system-based ion source was used to bombard a flat metal target, consisting of high refractive index (HI) and low refractive-index (LO) materials at a set angle of incidence of 57°. Typical deposition speeds for low and high refractive indexes were 1 Å/s and 0.6 Å/s, respectively. High and low index targets were swapped with a linear translation stage. The substrates holding the circular rack was rotated around its axis at an approximate speed of 20 rpm.

**Figure 1:**
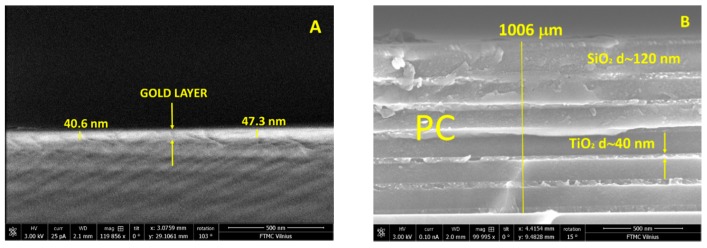
SEM micrographs of the surface plasmon polariton (SPP) sample (**A**) and six bilayers of ~120 nm SiO_2_ and ~40 nm TiO_2_ and 40 nm of TiO_2_ layer on the top of the Bloch surface wave (BSW) sample (**B**).

The structures of gold layer and PC were characterized by using scattering electron microscopy (SEM), dual beam Helios Nanolab 650 (FEI) (Oxford Instruments, Abingdon, UK). SEM studies of microstructure have shown that gold layer thickness on the glass substrate was about 43 nm, meanwhile PC consisted from six bilayers with TiO_2_ d = 40 nm and SiO_2_ d = 120 nm and 40 nm thickness of Ti_2_O layer on the top (Figure 1A,B).

**Figure 2:**
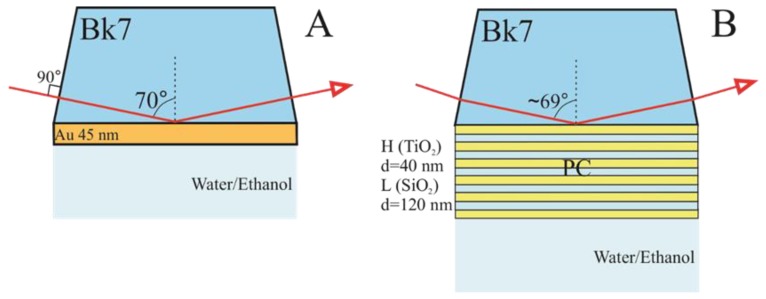
Samples structures and measurements configurations for SPP (**A**) and for BSW (**B**) excitations.

The experimental setup (Figure 2A,B) used for these investigations consisted of a dual rotating compensator ellipsometer RC2 (J. A. Woollam Co., Inc., Lincoln, NE, USA. The spectroscopic ellipsometry experiments were carried out in the 210 nm–1700 nm spectral range. The commercial SPR chip (Xantec, Dusseldorf, Germany) consisting of 1 mm thick BK7 glass slide covered with a gold film of thickness of about 45 nm was connected with a BK7 (70°) glass prism through the BK7 refractive index matching liquid (Cargille, Cedar Grove, NY, USA) to obtain optical contact between these elements. Further, a glass prism with an SPR chip was fixed into a custom-made Teflon chamber filled with deionized water or pure ethanol.

The structures supporting BSWs, distributed Bragg gratings were formed on the tops of the substrates (BK7 glass substrate), which had six bilayers of ~120 nm SiO_2_ and ~40 nm TiO_2_ and 40 nm of TiO_2_ layer on the top. For the excitation of BSWs the PC structure then was attached to the base of the BK7 prism through the optical contact as mentioned above. For both samples with the supported BSW and SPP modes, the experiments were conducted in a TIRE configuration. These used a glass BK7 prism (70°) at AOI of 70° (Figure 2A) for SPP and 68° (Figure 2B) for BSW. In case of BSW excitation optical scheme, the angle of incidence was 68° for better optimization of resonance. So, according to Snell’s law the internal angle of incidence in the prism was about 69.3°. The liquid handling system with a custom-built Teflon chamber was used in which the surfaces of both samples of SPP and BSW were placed. The chamber was filled with deionized water, which as then changed to ethanol, whose refractive index is higher than water.

The measured experimental data was then analyzed using the data acquisition software CompleteEase (J.A. Woollam Co., Inc., Lincoln, NE, USA) in a multi-layer model. The analyzed system for the BSW sample consisted of the BK7 glass prism, six bilayers of SiO_2_/TiO_2_ (120 nm/40 nm)/TiO_2_ (40 nm) and deionized water or ethanol. The SPP multi-layer optical model consisted of the BK7 glass prism/a gold layer (45 nm) and deionized water and ethanol. The thicknesses values of the gold layer for SPP and TiO_2_/SiO_2_ for bilayers in PC were taken according to the results obtained from SEM data. For the gold layer and for the PC in ionized water, simulated data were fitted to the experimental TIRE results with the mean square error values MSE = 4.1 for SPP and MSE = 7.6 for BSW, respectively.

## 3. Results and Discussions

**Figure 3:**
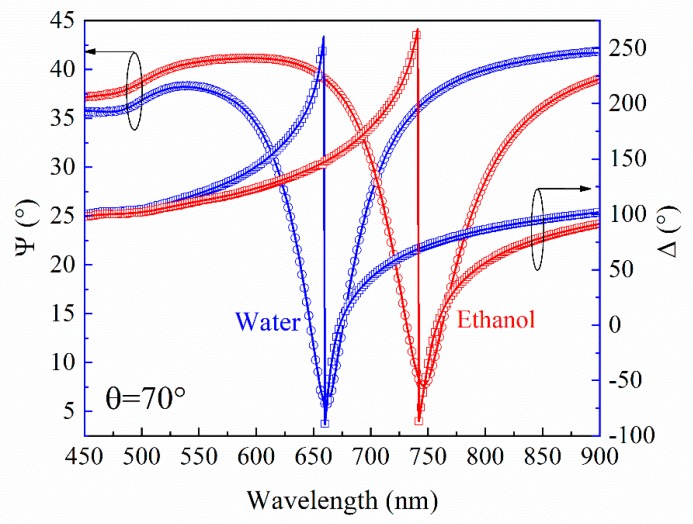
Experimental (dots) and calculated (solid curves) spectra of SPP sample before (blue curves) and after (red curves) deionized water changed to pure ethanol. Angle of incidence *θ* was 70 degrees.

**Figure 4:**
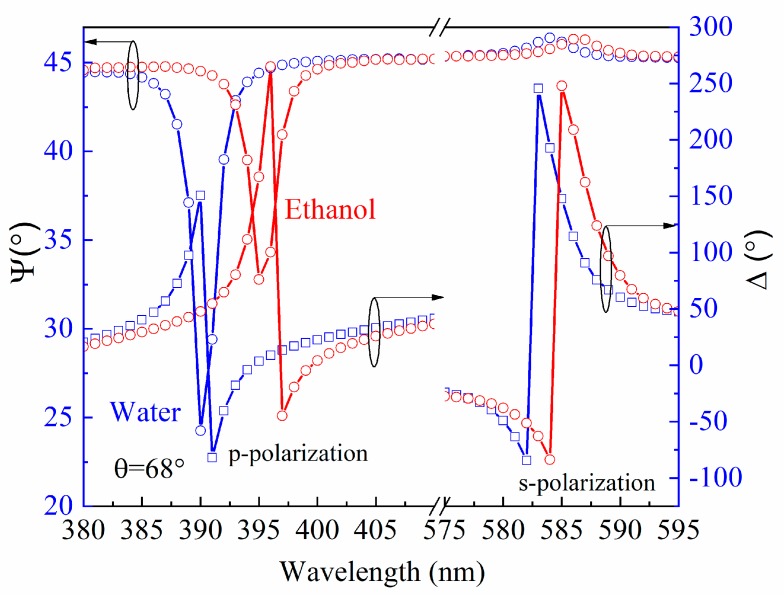
Experimental (dots) and calculated (solid curves) spectra of BSW sample before (blue curves) and after (red curves) deionized water changed to pure ethanol. Angle of incidence *θ* was 68 degree for better optimization of optical response.

It is shown by means of the ellipsometry method in its TIR configuration, that better sensitivity could be achieved due to phase registration, than with intensity detection for both SPP and BSW [21]. Moreover, the detailed analysis of the polarization properties give wide possibilities for studies of the optical responses, especially for the BSW excitations, where the BSW is not always clearly detectable from the reflection intensity spectra. In the case when the coupling is too strong (insufficient thickness of the PC or at very low values of the extinction coefficient k in the alternating layers), the dip in the reflection spectra is miniscule or even does not appear [25]. However, this does not influence the abrupt phase changes of the ellipsometric parameter *Δ* (*λ*) in the vicinity of the BSW resonance. Because of this, the BSW can always be detected in the ellipsometric scheme. Thus, the TIRE measurements were performed for the BSW supported sample and afterwards for the SPP in a water solution for both, which is the typical environment for studies of bio-interactions. For the plasmon active sample, the SPP resonances manifested themselves as dips in the *Ψ* (*λ*) and abrupt changes in the *Δ* (*λ*) spectra at 661 nm (Figure 3). In the case of the sample, which supported the BSW, the excitations were monitored at 390 nm and 584 nm for TM- and TE-polarizations, respectively (Figure 4). After the change of the deionized water to pure ethanol, the ellipsometric parameters *Ψ* and *Δ* red-shifted to 746 nm for the SPP.

Meanwhile for the BSW, the shift in the spectra was significantly smaller, 4 nm up to 394 nm for TM-polarization and 2 nm up to 586 nm for TE-polarization. The predicted behavior of the optical responses of both excitations was determined by the changes of the refractive index from pure water to ethanol. As can be seen from Figure 3 and Figure 4, the changes in the ellipsometric parameters *Ψ* and *Δ* due to the increase of the refractive index of the semi-infinite medium were *δΨ* = 33.8°, *δΔ* = 201.6° in the SPR case, while for the BSW case, these values were *δΨ* = 22°, *δΔ* = 167°, *δΨ* = 0.45°, *δΔ* = 77.2° for the TM- and TE-polarizations, respectively. The deionized water and ethanol dispersions of the refractive indexes were evaluated from the TIRE measurements where the prism was connected with the chamber for liquid handling and further, from the regression analysis. The Cauchy function was used for the refractive index dispersion of the semi-infinite medium and the optical constants were very close to those, which are found in the literature [26,27]. Further, the optical constants of the materials being used, namely the BK7 [28], were SiO_2_ [29], TiO_2_ [30] and Au [31].

**Figure 5:**
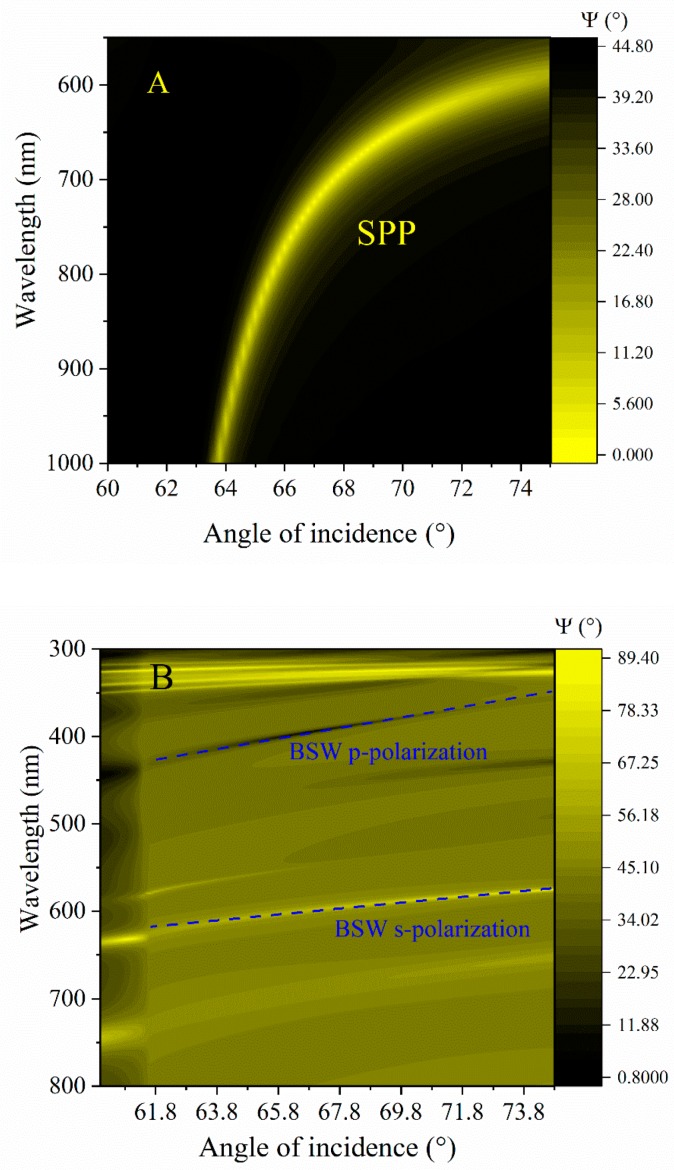
Numerical calculation of dependence of the resonance wavelength shift over the angle of incidence for SPP (**A**) and for BSW (**B**). The parameters of the model structures for both cases were the same as in the experiment.

It is thus reasonable to assume that such differences in the optical responses between the SPP and BSW were caused by the different dispersion features. Numerical calculations were performed in order to reveal the differences in sensitivity for SPP and BSW (Figure 5 A,B). The parameters of the model structures for both cases were the same as in the experiment. The simulation results clearly showed a much stronger dependence of the resonance wavelength shift over the angle of incidence for SPP than for BSW. In Figure 5A the yellow curve represents the optical dispersion properties of SPP, meanwhile in Figure 5B dashed blue lines indicates both BSW excitations for p- and s-polarizations.

In order to reveal the origins of the sensitivity features of the BSW and SPP, an analysis of the dispersion relations of both surface excitations was conducted. Many similarities between BSW and SPP can be found, such as the same general form of both excited surface electromagnetic waves, which are described by the same expression of propagation constant (Equation (1)). The excitation geometries for both BSW and SPP require one to match the in-plane wave vectors, which are longer than the light wave vector in a vacuum, thus couplers should be used (prism, grating, waveguide or others). BSW and SPP are evanescent surface waves and from the Maxwell’s equations under appropriate boundary conditions, the general expression of dispersion relation is the same for both surface excitations [5,32]:

(1) $$\beta =k_{p}\sqrt{\frac{\varepsilon_{1}\varepsilon_{2}}{\varepsilon_{1}+\varepsilon_{2}}}$$

where *β* is the BSW or SPP propagation constant, $k_{p}$ is the coupler wave vector (prism for our case) $k_{p}=\frac{2\pi }{\lambda }n_{p}sin\theta$, $\varepsilon_{1}$ is the relative permittivity of the PC dielectric layer at the interface (BSW) or metal layer in the case of SPP excitation, $\varepsilon_{2}$ is the relative permittivity of external dielectric medium (air or liquids), *λ* is the resonance wavelength for BSW or SPP, $n_{p}$ is the prism refractive index at a given wavelength, θ is the angle of incidence in the prism.

However, the main differences between these excitations come from the various interfaces. In the case of the SPP, these are from the metal/dielectric and for the BSW, from the dielectric (PC)/dielectric. For the resonance conditions of SPP, the real part of relative permittivity $\varepsilon_{1}$ must be real and have a negative sign:

(2a) $$\beta_{SPP}=k_{p}\sqrt{\frac{\varepsilon_{1}^{\prime }\varepsilon_{2}}{\varepsilon_{1}^{\prime }+\varepsilon_{2}}}$$

where $\varepsilon_{1}^{\prime }$ is the real part of complex dielectric permittivity of metals.

Meanwhile for the BSW, both materials are dielectrics with positive values. The dispersion relation is expressed as:

(2b) $$\beta_{BSW}=k_{p}\sqrt{\frac{\varepsilon_{1}\varepsilon_{2}}{\varepsilon_{1}+\varepsilon_{2}}}$$

where $\varepsilon_{1}$ and $\varepsilon_{2}$ are the relative permittivities of the top PC dielectric layers at the interface and the external dielectric medium (air or liquids), respectively. This fact has a main contribution to the dispersion laws and as a result, determines the sensitivity features of SPP and BSW. The large negative real part of the complex dielectric permittivity of metals in the term $\sqrt{\frac{\varepsilon_{1}^{\prime }\varepsilon_{2}}{\varepsilon_{1}^{\prime }+\varepsilon_{2}}}$ of the experession (2a) determines the bigger changes of the propagation constant $\delta \beta_{SPP}$ for SPP than the $\delta \beta_{BSW}$ for BSW because the perturbations of the dielectric permittivity at the interface of the two dielectrics are always smaller (in UV–VIS range) than for the metal–dielectric interfaces. For the real experimental conditions where the BSW is excited (for bio-sensing), the formed structures on the sensor surface usually do not exceed the value of the dielectric permittivity *ε* = 7.5 (*λ* = 390 nm) [28] for the titanium oxide layer, which indicates that changes of $\varepsilon_{}$ can be from 1.77 (aqueous medium) up to ≈8 (TiO_2_, Hf_2_O or similar). Meanwhile for the metal–dielectric interface, the real part $\varepsilon_{1}^{\prime }$ of the dielectric permittivity has a value of ≈−15 for gold (*λ* = 661 nm) in the vicinity of the SPP resonance. As a result, the changes of the propagation constants are δβSPP=1.2×105 radm and δβBSW=4.8×103 radm, respectively. This gives $\frac{\delta \beta_{SPP}}{\delta \beta_{BSW}}=25$ factor better sensitivity for the SPP than for the BSW. Indeed, this can be easily predicted from both the resonance shifts in the spectra, which were 90 nm for SPP and 4 nm for the BSW, respectively. Such differences in the changes of the propagation constants due to the perturbation of the dielectric permittivity’s produce $\lambda_{SPP}=2951\;nm/RIU$ and $\lambda_{BSW}=171\;nm/RIU$ sensitivities for SPP and BSW, respectively.

However, it was shown that the low losses in the dielectrics gave some advantages for the BSW and can be comparable with the SPP in bio-sensing applications [3,4,16]. This increased sensitivity was achieved in the BSW based sensors when a high refractive index material was used as the top layer of the PC. The structures of the PC were produced from alternating dielectric layers with very low extinction coefficient k, in order to optimize the BSW resonance, which becomes very sensitive to any materials with losses [4]. Low losses in dielectrics give significantly narrower widths of the resonance dip for *Ψ* and abrupt changes of the *Δ* ellipsometric parameters, which partially compensate for the much smaller spectral shift and lead to comparable sensitivity with SPP when monitoring the *Ψ* and *Δ* ellipsometric parameters, instead of the spectral shift.

As noted above, the experimental TIRE studies have shown that due to the increase of the refractive index by switching from water to ethanol, $\delta n_{\left(\lambda =661nm\right)}=1.3597-1.3309=0.0288$ for SPP and $\delta n_{\left(\lambda =390nm\right)}=1.3733-1.3597=0.0292$ for the BSW ellipsometric parameters changes. A sensitivity of ellipsometric parameters *δΨ/δn* (°/RIU) and *δΔ/δn* (°/RIU) and resonant wavelengths *δλ_res_/δn* (nm/RIU) could then be calculated for SPP and BSW cases. The measured changes of ellipsometric parameters and evaluated sensitivities and resolutions for the SPP and the BSW (for TM-polarization, because it was more sensitive) are summarized in the Table 1.

The resolution of the commercial J.A. Woollam RC2 spectroscopic ellipsometer was $\Psi =45^{{}^{\circ}}\pm 0.02^{{}^{\circ}}$ and $\Delta =0^{{}^{\circ}}\pm 0.05^{{}^{\circ}}$. As can be seen from Table 1, the sensitivity of the ellipsometric parameters *Ψ* and *Δ* were comparable for SPP and BSW configurations with slightly more sensitivity for the SPP. For the *Ψ* parameter, the SPP in ellipsometric parameter *Ψ* was more sensitive than the BSW by a factor of 1.56 and for *Δ*, this ratio was 1.22. As mentioned before, the resonance wavelength scanning measurements produced the largest differences in sensitivity between the SPP and BSW optical responses, in favor of the former. It should be noted that the ellipsometric parameter *Δ* is of special interest because of its higher sensitivity than *Ψ* for both excitations of SPP and BSW. Moreover, in the case of an ideal 1D PC (extinction coefficient k = 0) or with optimized low k 1D PC substrates, the dip of *Ψ* in the excitation point of BSW is very small or even disappears [25]. However, *Δ* is always clearly detectable with the same abrupt amplitude and wide linear shift in the spectra.

## 4. Conclusions

Summarizing, the TIRE method was used for the excitation and study of the sensitivity features of SPP and BSW resonances. Numerical calculations of the optical dispersions and the experimental TIRE data showed that SPP resonance overtake the BSW in wavelength scanning by a factor of about 17. However, for the ellipsometric parameters *Ψ* and *Δ* in the vicinity of excitations, the BSW sensitivity was comparable with SPP. The SPP was more sensitive by a factor of 1.56 for *Ψ* and a 1.22 factor for *Δ*. These studies showed that SPP sensitivity was still very high, however, BSW excitations had some advantages and sensitivity comparable with SPP could be achieved. Phase measurement should be performed in order to achieve the highest possible sensitivity and linear signal responses in BSW based optical sensors. Together with better biocompatibility of the various dielectric materials, BSW based biosensors are a promising platform for advanced optical sensing. Moreover, the longer propagation length due to low losses in the dielectric structures supported BSW open wide possibilities not only for optical sensing, but also for photonic devices for optoelectronic integrated circuits in a wide spectral range from VIS up to the THz region.

## Figures and Tables

**Table 1:** Comparison of SPP and BSW sensors sensitivity in total internal reflection ellipsometry (TIRE) configuration.

	*δΨ_SPP_*	*δ* *Δ* *_SPP_*	*δΨ_BSW_*	*δ* *Δ* *_BSW_*
*δ* (°)	33.8	201.6	22	167
Sensitivity (°/RIU)	1174	7000	735	5719
Resolution (RIU)	$1.7\times 10^{-5}$	$7.14\times 10^{-6}$	$2.7\times 10^{-5}$	$8.7\times 10^{-6}$
